# Recent Advances in Stimuli-Responsive Doxorubicin Delivery Systems for Liver Cancer Therapy

**DOI:** 10.3390/polym14235249

**Published:** 2022-12-01

**Authors:** Elena Ruxandra Radu, Augustin Semenescu, Stefan Ioan Voicu

**Affiliations:** 1 Department of Analytical Chemistry and Environmental Engineering, University Politehnica of Bucharest, 011061 Bucharest, Romania; 2 Advanced Polymers Materials Group, University Politehnica of Bucharest, 011061 Bucharest, Romania; 3 Faculty of Materials Science, University Politehnica of Bucharest, Splaiul Independentei 313, 060042 Bucharest, Romania; 4 Academy of Romanian Scientists, Splaiul Independentei 54, 030167 Bucharest, Romania

**Keywords:** doxorubicin, liver cancer, smart drug delivery systems, stimuli-responsive polymers

## Abstract

Doxorubicin (DOX) is one of the most commonly used drugs in liver cancer. Unfortunately, the traditional chemotherapy with DOX presents many limitations, such as a systematic release of DOX, affecting both tumor tissue and healthy tissue, leading to the apparition of many side effects, multidrug resistance (MDR), and poor water solubility. Furthermore, drug delivery systems’ responsiveness has been intensively studied according to the influence of different internal and external stimuli on the efficiency of therapeutic drugs. In this review, we discuss both internal stimuli-responsive drug-delivery systems, such as redox, pH and temperature variation, and external stimuli-responsive drug-delivery systems, such as the application of magnetic, photo-thermal, and electrical stimuli, for the controlled release of Doxorubicin in liver cancer therapy, along with the future perspectives of these smart delivery systems in liver cancer therapy.

## 1. Introduction

Liver cancer is one of the most common types of cancer, ranking in the top five in incidence with up to 1,000,000 cases reported per year worldwide [1]. From an etiological point of view, the main risk factors that favor liver cancer are viral infection with Hepatitis B and Hepatitis C, liver cirrhosis, obesity, and type II diabetes [2,3]. There is information linking the increased incidence of developing liver cancer by up to 50% in patients with cirrhosis [4]. Along with liver cancer, cirrhosis results from a repercussion of different conditions, such as chronic viral hepatitis, alcohol intake, fatty liver disease, and other liver dysfunction [5,6]. The principal malignancy of the liver accounting for more than 5% of all types of liver cancers that occurs predominantly in patients with underlying chronic liver disease such as cirrhosis is hepatocellular carcinoma (HCC) [7]. Unfortunately, liver cancer is most often diagnosed in the terminal phase because the early stages have an asymptomatic nature [3,8,9]. The average life expectancy of patients without treatment is 1–3 months and for patients with treatment is 6–20 months [1,10]. The most used method of treating cancer is to surgically remove the cancerous tissue, but in the case of liver cancer, the survival rate is 47% to 53% [8]. In addition, chemotherapy and radiotherapy represent other alternatives as cancer treatments [9]. Chemotherapy is a mandatory part of clinical cancer treatment, through the administration of antitumoral drugs in order to inhibit the growth of the tumoral cells and generate cellular apoptosis. The recently reported studies have shown poor results correlated with chemotherapy [9]. Unfortunately, the administration of anticancer agents is performed systemically, and the antitumor agent can affect both tumor cells and healthy cells. This can cause both side effects and poor treatment effectiveness due to the off-target phagocytic uptake and nonspecific biodistribution of the drugs [11]. Another disadvantage of chemotherapy is multidrug resistance (MDR), which is closely related to tumor recurrence and therapeutic failure, and poor water-solubility [10,12]. The most used antitumoral agent in the chemotherapy treatment for HCC is Doxorubicin (DOX), along with chemotherapeutic drugs, such as 5-fluorouracil and cisplatin [9,13,14].

**Figure 1:**
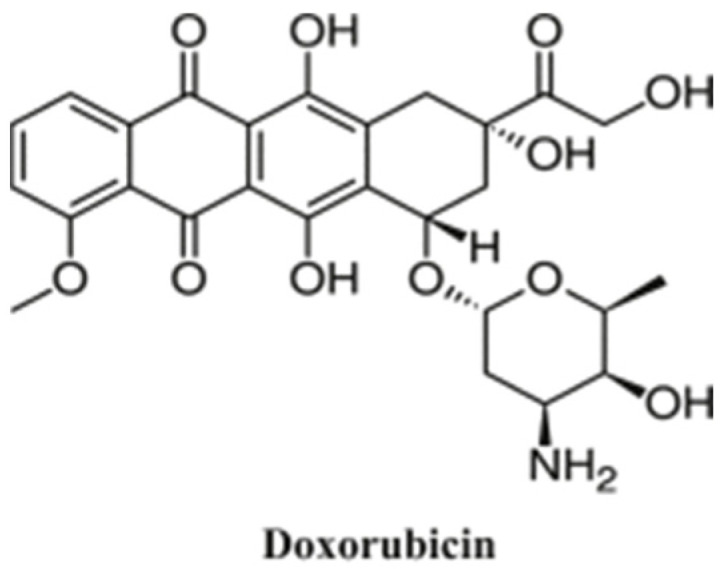
Chemical structure of DOX (Reprinted with permission from Ref. [18]. 2019, Gopalakrishna Pillai).

Doxorubicin (DOX) (Figure 1), isolated from *Streptomyces peucetius*, is one of the most commonly used drugs in cancer treatment, including breast, bile ducts, prostate, uterus, ovary, esophagus, stomach, and liver cancer [15,16,17,18]. DOX was one of the first antitumoral agents used in liver cancer treatment [5]. It was reported that the antitumor activity of doxorubicin is due to its ability to intercalate into the DNA helix and/or bind covalently to proteins involved in DNA replication and transcription, resulting in an inhibitory effect on DNA, RNA, and proteins’ synthesis, leading to cell death [19,20]. However, the limitations of DOX are drug resistance and the side effects, mainly because of its toxicity on non-cancerous cells. The main side effects of the usage of DOX are nausea, vomiting, myelosuppression, and arrhythmia in a very short time after administration [21]. Unfortunately, this cellular damage caused by DOX administration not only occurs in cancer cells but also in healthy cells, such as a DNA alteration caused by the presence of Adriamycin, which leads to a slowing or stopping of the cells’ growth [7,21]. Furthermore, studies have shown that the use of DOX in conventional chemotherapy can lead to cardiotoxicity through increased oxidative stress, affecting the heart tissue and leading to cardiomyopathy [22,23]. Nowadays, researchers are developing new forms of drugs able to optimize the pharmacodynamics and pharmacokinetics that specially target the tumor tissue, without affecting the surrounding healthy tissues [24,25].

Materials science with its interdisciplinary character has provided a lot of solutions in the field of biomedical sciences for different applications in recent decades, solving a lot of problems that were previously impossible to address. In the twentieth century, membranes for hemodialysis appeared for kidney failure disease [26,27], a large number of materials were discovered for tissue engineering from soft materials [28,29,30] to composites for bone repair and regeneration [31,32], or even solutions that minimize the impact of the health system through the environment and everyday life in terms of waste. One of the applications that attracted the interest of researchers in the field of materials for drug delivery.

In the hopes of solving the problem related to the efficiency of cancer treatment, various drug delivery systems (DDSs) have been researched in the past few decades, aiming at targeted delivery and controlled release of antitumor agents [33,34,35,36,37,38,39]. The principal interest is to develop new drug delivery systems to guarantee a safe administration of the therapeutic agent, in order to improve the delivery efficiency, and not harm the human body by the presence of toxic side-effects [40,41]. The development of targeted release systems for cancer treatment may overpower the limitations of standard treatment, such as low stability, fast inactivation or degradation in vivo, non-specific toxicity, poor solubility, unfavorable pharmacokinetics, and low biodistribution [42]. A multitude of controlled and targeted release systems have been reported in the literature, based on the use of organic [43] or inorganic [44,45] nanoparticles, polymeric micelles [46], dendrimers [47], nano shells [48], and nanotubes [49]. Smart drug delivery systems are able to respond to a range of diverse stimuli, such as internal stimuli (pH, redox conditions, enzymatic activity, the concentration of specific biomolecules, temperature), or external stimuli (such as applying magnetic fields, electric fields, ultrasound, mechanical pressure, etc.) [50]. Additionally, many reviews in the domain of liver cancer therapy are continuously published due to the extensive interest in the subject. Some of the latest topics are related to the use of nanoparticles and a multitude of active pharmaceutical compounds [51,52,53,54], the nature of materials used for different types of technological solutions for cancer problems related to drug delivery [55,56,57], or medical aspects of liver cancer [58,59,60]. This review generally presents the main recent trends in the obtaining of smart drug-delivery systems field which are internal or external stimuli-responsive for the efficient release of DOX for the treatment of hepatocellular carcinoma. These intelligent release systems are obtained in particular from polymeric materials, which have the role of facilitating the controlled and targeted release of Doxorubicin, reducing systemic toxicity, increasing drug bioavailability, reducing and preventing the many side effects of Doxorubicin, improving selectivity, biocompatibility, dispersibility and the stability of Doxorubicin. A summary of the reviewed studies in tabulated form is presented at the conclusion (Table 1).

## 2. Internal Stimuli-Responsive Drug Delivery System

### 2.1. pH-Responsive Drug Delivery Systems

The variation of pH values in different regions of the human body can provide a suitable physiological stimulus for pH-responsive drug delivery in order to deliver the active substances in the area of interest [82]. For example, the pH range of the stomach is between 1.5–3.5 pH, 5.5–6.8 pH of the intestine, 6.4–7 pH of the colon, and up to 7.4 pH of the blood [83]. The pH of cancerous tissue exhibits a decreasing trend due to the Warburg effect, which explained that the hypoxic cells produce lactic acid due to glycolysis [35]. Further, pH-responsive drug delivery systems have excellent advantages and have attracted much attention in the past decade because the pH values in tumors and inflammatory tissues are significantly lower than those in blood and normal tissues and could increase the therapeutic efficacy of administrated drugs [33,35,84,85,86]. The main principle of pH-responsive drug delivery systems is to release the active substance when the pH trigger point is achieved, and the intracellular concentration of drugs is equal to the therapeutic dose needed [34]. This release system time has the role of releasing the active substance preferentially, in order to eliminate the disadvantages of chemotherapy treatment.

One modality to obtain this type of release system is to introduce “ionizable” chemical groups, for example, amines, phosphoric acids, and carboxylic acids among others, with nanomaterials [87]. These groups, with varying pKa values and chemical structures, have the ability to accept or donate protons and be subjected to pH-dependent alterations in the chemical or physical properties, such as solubility and swelling ratio, culminating in drug release [34]. Various biomaterials have been reported in the obtaining of pH-responsive drug delivery systems, such as inorganic nanoparticles, core-shell nanoparticles, liposomes, hydrogels, and polymer micelles [12,88,89,90]. An example of some widely used materials for the production of pH-sensitive drug delivery systems are pH-sensitive polymers, which are polyelectrolytes with ionizable groups in their backbones, side groups, or end groups. When the pH of an aqueous solution changes, these pH-sensitive polymers are ionized, resulting in a change in their conformation. These “smart” polymers can either accept or donate H+ ions in response to the pH changes in the environment. The protonation or deprotonation of these ionizable groups can lead to changes in the structure of the polymer chain by electrostatic repulsion of the generated charges, which causes the transition of the chains from collapsed to an expanded state [83]. For example, pH induces protonation/deprotonation in the -NH2 groups of chitosan, making it susceptible to use in obtaining pH-sensitive release systems [91,92]. At acidic pH (<6), primary amines are protonated and positively charged, making chitosan soluble in an aqueous solution [93]. In addition to sensitivity to pH, chitosan is low toxicity, biocompatible, has antibacterial properties, mucoadhesive properties, and permeation-enhancing effects [94,95]. Due to these beneficial characteristics of chitosan, many researchers have used chitosan in various pH-sensitive release systems for liver cancer [96,97,98,99,100]. Mi et al. [96] reported pH-sensitive drug delivery for DOX based on Carboxymethyl-β-Cyclodextrin/Chitosan Nanoparticles. In vitro release showed higher cumulative release rates of DOX from HF-DOX-CD NPs at a low pH, than cumulative release at neutral pH or slightly basic pH due to the tendency of chitosan nanoparticles to swell at low pH, and also, due to the acidic pH, the protons might penetrate the interior and attack the inner secondary bonds of the nanoparticle. These two reasons lead to a release of DOX in an acidic medium. Zhan et al. [79] developed pH-sensitive and self-healing properties based on 4armPEGDA and N-carboxyethyl chitosan for liver cancer. The degradation rate was observed to be higher in the case of exposure to an acidic environment indicating that hydrogels have pH-dependent degradation behavior. Cytotoxicity tests were performed on human hepatocytes (L02) and the therapeutic effect of DOX-loaded CEC/4armPEGDA hydrogels on HepG2 cells. The results showed no toxicity for human hepatocyte cells and even at a fairly small load of DOX the obtained system was able to kill tumor cells over time due to the continuous release of DOX in the hydrogel during this process. Qu et al. [65] presented the obtaining of a pH-sensitive hydrogel that was able to release DOX into an acidic environment based on N-carboxyethyl chitosan (CEC) and dibenzaldehyde-terminated poly(ethylene glycol) (PEGDA) as a possible treatment for hepatocellular carcinoma. At an acidic pH, the amino groups of chitosan become protonated and positively charged, resulting in weaker -NH_2_ and -CHO bonds, that also decompose and the release of the DOX takes place.

**Figure 2:**
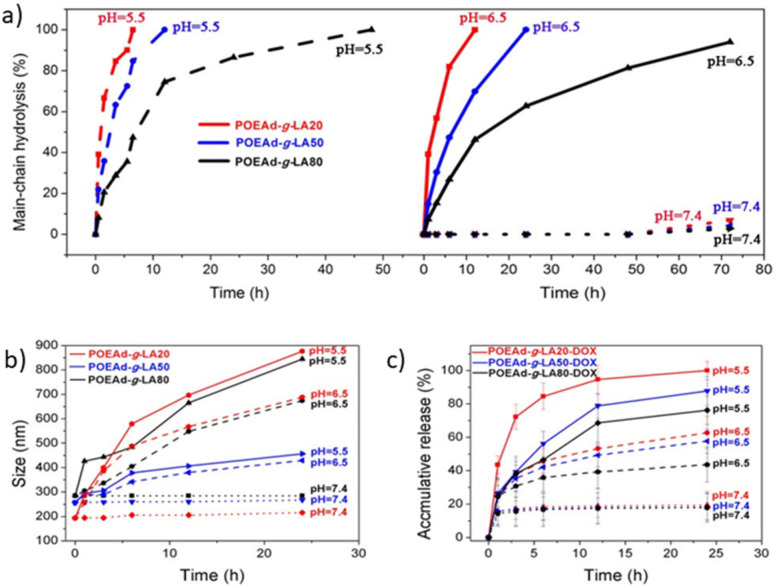
(**a**) Hydrolysis kinetics of POEAd-g-LA micelles at pH 7.4, 6.5 and 5.5; (**b**) Time and pH-dependent changes of average size of POEAd-g-LA micelles in aqueous phosphate buffer measured by DLS; (**c**) Release kinetics of loaded DOX in POEAd-g-LA micelles at pH 7.4, 6.5, and 5.5 (Reprinted with permission from Ref. [61]. 2017, Guoqing Yan).

Furthermore, many polymeric networks have been studied for showing pH-sensitive bioerodible properties such as Poly(Ortho Ester Amides) (POEAd) due to the hydrolysis of ortho esters at low pH [61]. Yan et al. [101] proposed a solution for the promotion of drug accumulation and efficient killing ability of tumoral cells via galactose-grafted on an ultra-pH-sensitive drug carrier (POEAd-g-LA-DOX micelles), which can respond to both intracellular and extracellular pH, to remain stable at pH 7, responding to extracellular tumor pH, conjugating receptors in the cell membrane of liver cancer through surface galactose-ligands of micelles, and being sensitive to intracellular tumor pH following further swelling for rapid drug release. The POEAd-g-LA copolymers were successfully obtained via facile polycondensation followed by the grafting of lactobionic acid. Figure 2 shows the percentage of main chain hydrolysis over time, as well as size and accumulation release over time, at different pH values. In order to assess the influence of pH, Yan carried out an NMR analysis to follow the time-course of the hydrolysis of ortho esters in the main-chain for up to 72 h in deuterated water buffers (pH 7.4, 6.5, and 5.5). The hydrolysis of the ortho ester did not occur at neutral pH, while at pH 5.5 it was observed that the hydrolyzation of ortho esters was accelerated due to the acidic pH, which could lead to a size reduction and drug release. Furthermore, it was highlighted that the ultra-pH-sensitivity of ortho ester can influence the size, observing that at an acidic pH the size of the particles increases, swelling itself, which was probably due to the interaction between the hydrogen bonding interaction of the hydrophilic products with plenty of hydroxyl groups and amide, and gradually increasing hydration. Additionally, the rate of drug release was reported to be higher in the case of an acidic medium, which can thus be a benefit due to the acidic pH of the tumor tissue. The in vivo therapeutic efficacy of the reported formulations was tested in the mice bearing subcutaneous-inoculated H22 tumors, and it was observed that in relation to the mice treated with POEAD-g-LA20-DOX a decrease in the tumor size was reported, reducing the growth, and demonstrating an inhibitory effect on tumoral tissue.

**Figure 3:**
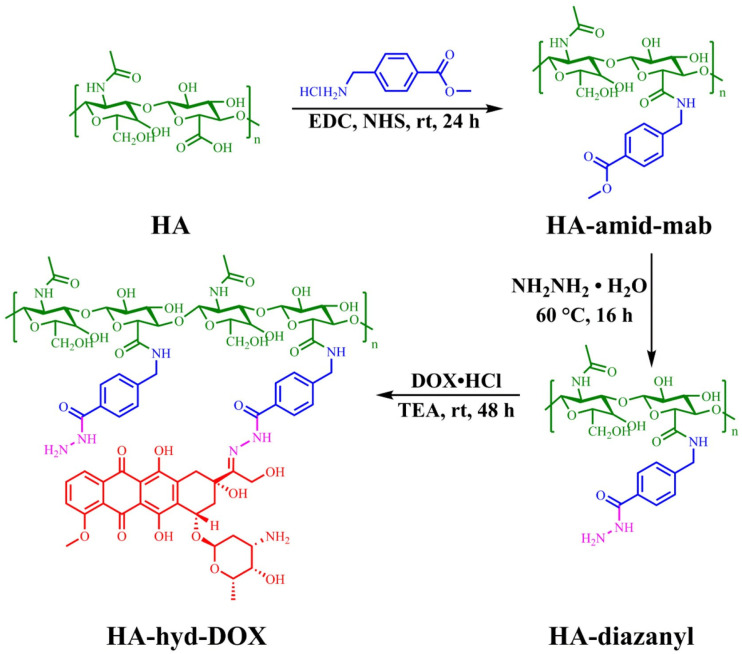
Synthetic routes of HA-hyd-DOX prodrugs. (Reprinted with permission from Ref. [62]. 2018, Jianhong Liao).

Hyaluronic acid (HA) has been widely used in targeted drug delivery systems due to its good biocompatibility, good water solubility, high selectivity, and affinity to CD44 receptors found in different tumor cells [101,102]. In order to increase the drug/cargo accumulation specifically in CD44 over-expressing cancer cells, HA-attached pharmaceuticals and nanocarriers have been created. This is because HA has numerous functional groups available for chemical conjugation with anticancer medications or nanocarriers of drugs or genes [103]. Further, it was reported that DOX could be covalently bounded on the backbone of HA through the hydrazone linkage [62,104]. Li et al. [105] described that hydrazone bonds disintegrated in lysosomal pH and remained stable at neutral pH. Liao et al. [62] reported a pH-responsive drug delivery of hyaluronic acid-hydrazone linkage-Doxorubicin (HA-hyd-DOX), which is illustrated in Figure 3. Due to the amphiphilic structure between the hydrophilic glucopyranose ring and hydrophobic DOX segment, a series of HA-hyd-DOX NPs were generated by self-assembling in an aqueous solution. The results showed a burst release in the first 6 h in buffers at pH 5.0 due to a looser structure of particles with the cleavage of hydrazone bonds at a lower pH. The cell cytotoxicity was studied on HeLa and L929 cells for 48 h of incubation and a nontoxicity against HeLa or L929 cells was observed, suggesting that HA is nontoxic, having good biocompatibility. Additionally, an internalization was observed due to the receptor-mediated binding affinity of CD44 for HA with high specificity.

**Figure 4:**
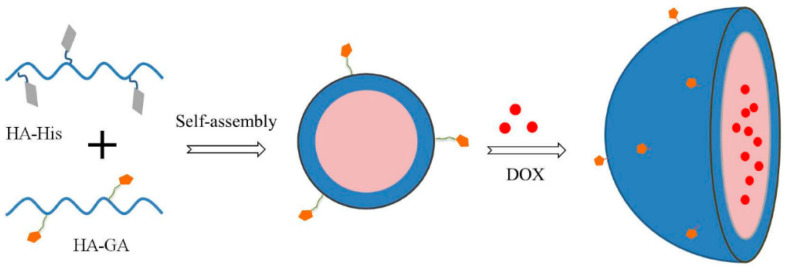
Preparation of DOX-loaded micelles (Reprinted with permission from Ref. [63]. 2016, Jing-Liang Wu).

Wu et al. [63] reported on the liver cancer-targeting mixed micelles based on hyaluronic acid–glycyrrhetinic acid conjugate and a hyaluronic acid-L-histidine conjugate S(HA–GA/HA–His) were prepared via ultrasonic dispersion, and the in vitro and in vivo investigation of antitumor effect of Doxorubicin (DOX)-loaded micelles (Figure 4). It was related to the pH sensibility of DOX-loaded HA–GA/HA–His is micelles and the remarkable absorption of HepG2 cells. It was reported that the hydrophobic DOX molecules were efficiently encapsulated into the HA–GA/HA–His micelles in an aqueous solution because of the presence of a hydrophobic core in the micelles. It was shown that the obtained DOX-loaded micelles exhibited a sustained DOX release under the acid pH of hepatocellular carcinoma cells, due to protonation of His, resulting in the swelling of the core, followed by the DOX release.

**Figure 5:**
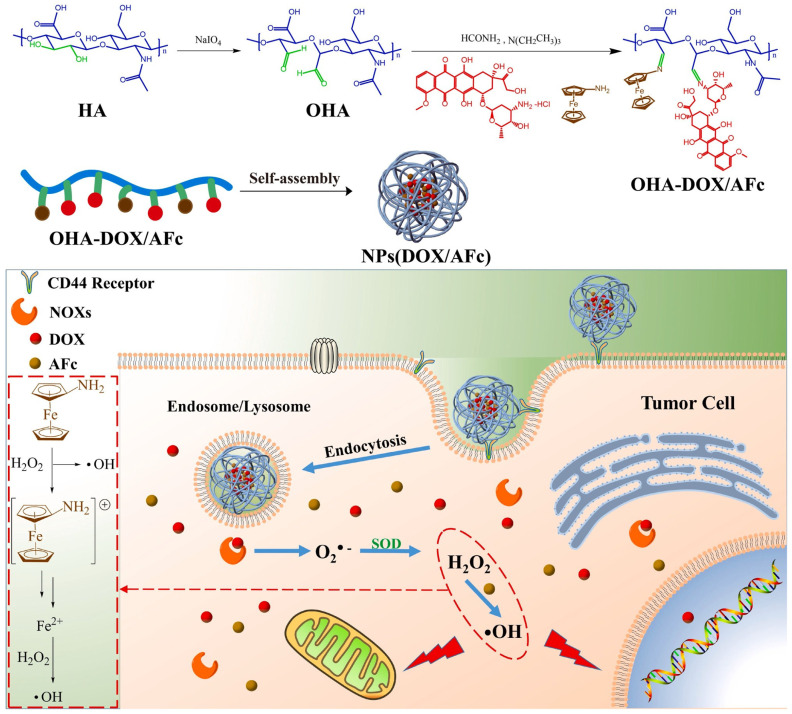
The schematic illustration of the synthetic routes of OHA-DOX/AFc prodrugs and the schematic diagram of NPs(DOX/AFc) entering tumor cells to play a combined therapeutic effect (Reprinted with permission from Ref. [106]. 2021, Mengheng Lei).

Anirudhan et al. [64] delivered DOX and cisplatin (CDDP) via pH-responsive drug delivery, also based on hyaluronic acid (HA) and chitosan (CS-NSA). The release was studied via immersion in two buffer media at pH 7.4 and 5.5 to mimic the intestinal fluid and tumor environment, at 37 °C, for 48 h. A better release of DOX was observed in comparison with cisplatin, for both pH levels. Further, a higher release was observed for both drugs at pH 5.5 (for CS-NSA/A-HA/DOX, the release was 89.0% at pH 5.5 and 42.0% at pH 7.4 and for CS-NSA/A-HA/CDDP, the release was 87.0% at pH 5.5 and 44.6% pH 7.4). Lei et al. [106] developed a pH-responsive drug delivery based on HA showed a great release under the endosomal/lysosomal environment for DOX (56.5%) (Figure 5).

Folic acid (FA) was reported as an effective targeting prodrug due to the selective binding of FA to the folate receptor (FR), which is overexpressed in cancer cells [107]. Zeolitic imidazolate framework (ZIF) coordination bonds are sensitive to low pH and this could be used for the delivery of an active substance, such as DOX [108]. Bi et al. [66] reported the delivery of DOX for human hepatocellular carcinoma via folic acid-modified and zeolitic imidazolate framework (ZIF) nanoparticles. It highlighted the pH sensitivity of the obtained nanocarrier; thus, it was observed that drug release of the drug delivery system based on folic acid is increased by the acidic environment. Further, it was reported that the release mechanism of the DOX is influenced by the ZIF degradation in acid environments and the increased solubility of DOX at lower pH as a result of increased protonation of amino groups in DOX molecules. In addition to the previously listed advantages of pH-sensitive systems, the limitations of pH-responsive drug delivery systems are that the pH level is an endogenous stimulus and this makes it difficult to control, a narrow range of pH variation, and steady kinetics for the drug release [39,109,110,111]. These limitations can be overcome by the development of multi-responsive drug delivery systems, so that the release is not based only on a single stimulus, such as pH.

### 2.2. Temperature-Responsive Drug Delivery Systems

One of the most closely studied stimuli of controlled release systems is temperature. Temperature-sensitive polymers are used in order to obtain this responsive DDS, for different biomedical applications, such as temperature-sensitive gels, liposomes, micelles, colloidal particles, mRNA recovery, and gene delivery [112,113,114]. These thermosensitive polymers are able to release the encapsulated active substance even at small temperature variations. Numerous thermosensitive compounds are used for obtaining thermoresponsive hydrogels in DDS applications, such as poly(N-isopro-pylacrylamide (PNIPAAm) derivatives, poly(ethylene oxide)-poly(propylene oxide) (PEO–PPO) pluronic copolymers), core–shell thermoresponsive NPs, polymeric nanotubes, polymeric micelles, layer-by-layer (LBL)-assembled nanocapsules, microbeads (MBs), and elastin-like polypeptides (ELPs) [50,115,116]. However, thermo-responsive release systems can retain the load at systemic circulation temperatures of 37 °C, but release a load rapidly when the temperature exceeds 40 °C, due to the locally heated tumor. These DDS experience a reversible phase transition from a molecularly dissolved hydrated state in an aqueous solution (hydrophilic) to a dehydrated state (hydrophobic) as a reaction to the slight variation in temperature leading to an induced sharp globule-to-coil conversion that generates the release of encapsulated antitumoral agents from these polymeric nanocarriers [117]. Depending on the critical solution temperature (CST), thermo-responsive polymers are classified into two categories: (i) polymers that have a low critical solution temperature (LCST), which means that these polymers are water-soluble and make homogenous systems below this temperature; and (ii) polymers that have an upper critical solution temperature (UCST), which means that these polymers are water-soluble and make homogenous systems above this temperature [117].

**Figure 6:**
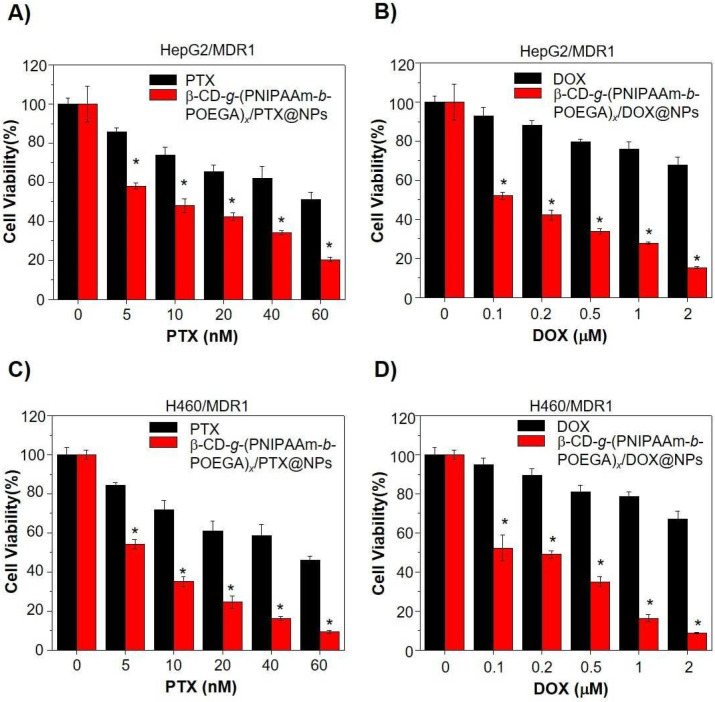
Cell viability assay to study β-CD-g-(PNIPAAm-b-POEGA)x/drug complex nanoparticles for reversing MDR1-related drug resistance. (**A**,**C**) Cell viability of (**A**) HepG2/MDR1 and (**C**) H460/MDR1 after 24 h treatment with indicated doses of PTX complex nanoparticles or equivalent PTX only. (**B**,**D**) Treatment with DOX complex nanoparticles or equivalent DOX only in (**B**) HepG2/MDR1 and (**D**) H460/MDR1 cell lines for 24 h. Data represented in the form of mean ± standard deviation. * *p* < 0.05, with comparison to growth inhibition of PTX or DOX treatment only, *n* = 6 (Reprinted with permission from Ref. [67]. 2018, Hongwei Cheng).

Poly(N-isopropyl acrylamide) (PNIPAA)-based materials show thermo–thermo responsiveness behavior which could be very useful in the development of different temperature-sensitive release systems [118]. Cheng et al. [67] developed a PEGylated star-shaped polymer based on the conjugation of the β-CD core with thermosensitive poly(N-isopropyl acrylamide) (PNIPAAm) and biocompatible poly(oligo(ethylene glycol) acrylate) (POEGA) arms in order to obtain temperature-sensitive drug delivery systems for liver cancer. The DOX was used as a model chemotherapeutic in order to study its in vitro cellular uptake. It was shown that only DOX and β-CD-g-(PNIPAAm-b-POEGA)x/DOX at 25 °C have a slow cellular uptake, but when the tempera ture was increased to 37 °C, a faster and higher cellular uptake was reported. Interestingly, at a temperature above LCST, PNIPAAm becomes hydrophobic, having a tendency to aggregate, forming the core of nanoparticles, meanwhile, the POEGA chains help maintain the integrity of the formed nanoparticles. In addition, it was reported at 80% in the first 6 h and 90% release after 24 h of DOX at 37 °C condition; meanwhile, at the 25 °C condition, a slow release of DOX was observed, where after 24 h, the release decreased to 53.9%. In Figure 6, the decrease in the cell viability is reported in the case of β-CD-g-(PNIPAAm-b-POEGA)x/DOX in comparison with neat DOX. The test was performed on HepG2 and H460 cancer cells. The same results were shown in the case of hydrophobic paclitaxel (PTX), which is a hydrophobic anticancer drug. Kunene et al. [68] reported pH and temperature-responsive based on magnetic graphene nanosheets (MGNSs), functionalized by poly(N-isopropylacrylamide) (PNIPAM) and polyethylenemine (PEI) nanogel for DOX delivery for liver cancer. The cell viability was reported at above 90% against HEK293 cells and HepG2 cancerous cells. The DOX release was reported higher at pH 5.4 than at pH 7.4 and above, the LCST was rapid, due to the swelling and de-swelling of the PNIPAM/ PEI nanogel at variations of the medium’s temperature.

Furthermore, other studies were reported in order to release DOX for liver cancer through temperature-responsive drug delivery. Mdlovu et al. [69] described a dual-responsive drug delivery system (pH- and thermo-responsive drug delivery system) based on magnetic iron oxide (MIO) nanoparticles functionalized with Pluronic F127 (PF127) and branched polyethylenimine (bPEI) and loaded with DOX. The DOX releases were dependent on temperature and pH as the highest release rate (54.8%) was in acidic conditions (pH 5.4) and when the temperature was increased from 37 °C to 42 °C an increased rate release (51%) was reported due to the LCST of the PF127 polymer which is 42 °C [119]. Furthermore, Mdlovu et al. [120] described pH- and thermo-sensitive Doxorubicin-conjugated magnetic SBA-15 mesoporous for hepatocellular carcinoma with a release rate of 70% in acidic conditions and 69% at 42 °C (pH = 7.4), which in comparison with previous work, showed an increase in the release values of DOX. Sebeke et al. [70] reported thermo-responsive drug delivery based on phosphatidylglycerol (DPPG2) via magnetic resonance-guided high-intensity focused ultrasound (MR-HIFU)-mediated hyperthermia. A rapid release was reported at 42 °C due to the melting temperature of DPPG2-TSL. Classical methods of chemotherapy agents are hemofiltration or plasma filtration [121,122]. Removal of ~30% of the administered dose was reported and a reduction in the toxicity of the remaining drug in the organism.

### 2.3. Redox-Responsive Drug Delivery Systems

At this moment, redox-responsive drug delivery systems have been intensely studied, to improve the controlled release of the antitumoral agents, via targeting the tumoral tissue through redox-response in the presence of glutathione (GSH) [71]. The cancerous tissue presents particular abnormal cellular environments, such as the presence of enzymes and reducing environments [123]. The main principle of redox-responsive drug delivery systems is employing the distinct differences in redox potentials between tumors and normal tissues. The reducing environment of cancerous tissue is based on the reduction and oxidation state of NADPH/NADP^+^ and glutathione (GSH, GSH/GSSG) [123,124]. The most popular redox couple is GSH/glutathione disulfide (GSSG) [125]. The GSH is a tripeptide of glutamate, cysteine, and glycine found at an increased concentration in ovarian, breast, lung cancer, head and neck cancer, and in lower concentration in brain and liver tumors compared to healthy tissue [126]. In addition, it was reported that the GSH plays an important role in cell differentiation, proliferation, and apoptosis, and imbalanced values may indicate cancer presence [127]. The GSH levels can be influenced by oxidative stress, and can act as a biomarker, to indicate the severity of cancer. The highest concentration of GSH values for healthy tissues is in the liver and hepatic GSH plays an important role in interorgan GSH homeostasis by being the main source of plasma GSH. Liver disease can decrease the concentration of GSH values due to multiple factors, such as reduction during oxidative stress, increased utilization and export, and decreased synthesis [128].

**Figure 7:**
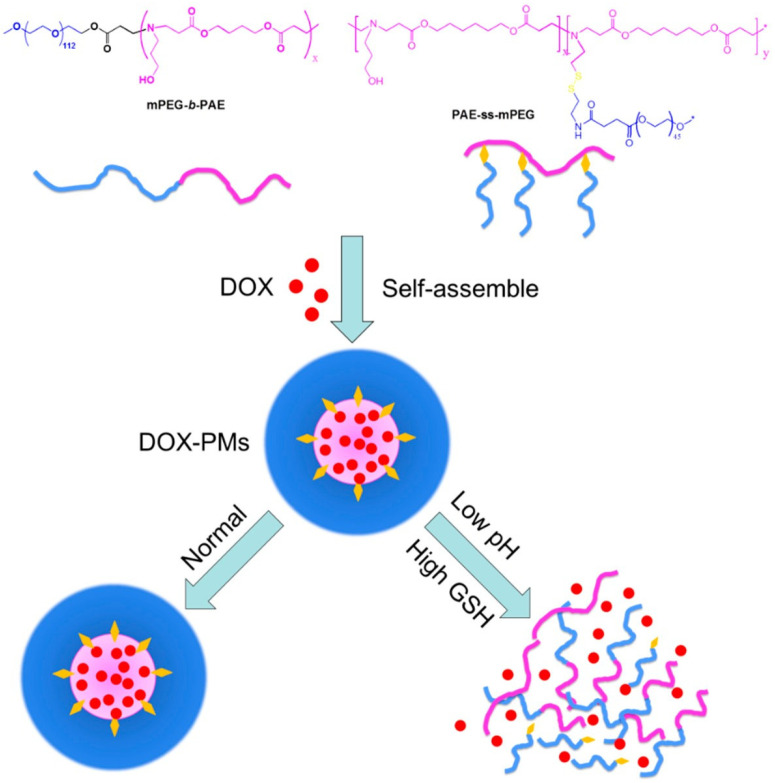
Co-micellization of pH/redox-responsive diblock copolymers for drug delivery and controlled release triggered by pH and glutathione (GSH). DOX: Doxorubicin; PMs: polymeric micelles. (Reprinted with permission from Ref. [131]. 2019, Yongle Luo).

The main advantages of redox-responsive delivery systems are the stability in contact with the normal tissue, which can decrease the systematic toxicity and side effects, the prompt response to high values of GSH concentration in tumoral cells to release the therapeutic agents, and the release of the therapeutic agent in the cytoplasm, which improves the therapeutic effect [123]. The main categories of redox-responsive drug delivery systems are disulfide bonds (which are able to break down via reducing glutathione to sulfhydryl group and break the drug delivery system and facilitate the therapeutic agent release) and diselenide bonds (the Se–Se bond and C–Se bond are with lower bond energy than that of the S–S bonds) [123,129,130]. Luo et al. [131] reported the pH and redox-responsive drug delivery for DOX via deprotonation/protonation under acidic pH and cleavage of disulfide bonds (Figure 7).

**Figure 8:**
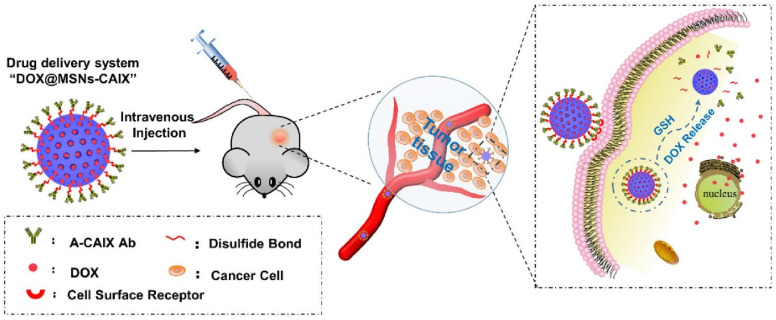
Illustration of A-CAIX Ab targeted mesoporous silica nanoparticles as a redox-responsive drug delivery system (Reprinted with permission from Ref. [71]. 2020, Minmin Chen).

These linkages express great stability in the oxidative extracellular medium, the therapeutic agent is released in the increased reductive intracellular compartments by thiol–sulfide exchange reactions [72]. Chen et al. [71] developed a new antibody-targeted and redox-responsive drug delivery system by binding the anti-carbonic anhydrase IX antibody (A-CAIX Ab) on the surface of mesoporous silica nanoparticles (MSNs) via disulfide linkages used as the vehicle to load the chemotherapy drug, Doxorubicin (DOX). In Figure 8, it is shown how the MSNs are used as a vehicle to load the DOX and CAIX grafted on MSNs by disulfide bonds. The in vivo tests performed on mice showed a reduction in tumor weight after only 11 days as well as MSNs loaded with DOX and DOX@MSNs-CAIX.

Wang et al. [132] developed a redox-responsive liposome, capable of probably leading both cancer stem cells and bulk cancer via the incorporation of Salinomycin (Sal), which is a hydrophobic drug, into the lipid layers of the obtained liposomes, and Doxorubicin (DOX), a hydrophilic drug, encapsulated into the aqueous cavity of the liposomes. It was shown that GSH potentially affects the disulfide bonds of obtained liposome lipid layers destabilizing the liposomal nanostructure and the presence of GSH may influence the fast release of DOX and Sel, leading to the synergistic inhibition of tumor growth and reduction in cancer stem cells’ stemness. Mezghani et al. [72] described that the introduction of disulfide linkages acts as a burst release constituent and the hydrophobic groups of the glycyrrhetic acid were covalently linked to the hydrophilic backbone of hyaluronic acid over amide bond development, with the mixture of an intermediate disulfide bond as a major component. The resulting swelled nanoparticles from the reduction in the disulfide bonds lead to hydrophobicity modification of the core of nanoparticles conducting to the production of aggregates, which can lead to the deconstruction of the nanoparticle in the deeply reductive environment. In addition, it was observed that the size of the nanoparticles remains unchanged in the absence of GSH, which indicates the stability of the obtained nanoparticles in the non-reductive media. Furthermore, the performed in vivo test showed that the obtained DDS accumulated in hepatic tissue, approving their targeting abilities.

**Figure 9:**
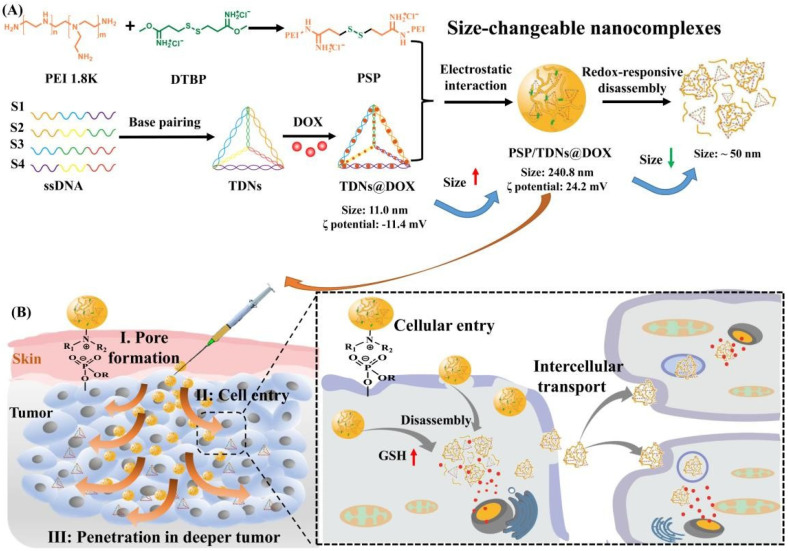
Schematic of the redox-responsive PEI/TDNs/DOX nanocomplexes with membrane-breaking and size-changeable properties for combating MDR tumors. (**A**) Synthesis and size-change mechanism of PSP/TDNs@DOX nanocomplexes. (**B**) Proposed process and underlying mechanism of PSP/TDNs@DOX nanocomplexes overcoming MDR via the formation of pore in plasma membranes, promoted cellular entry and cell-to-cell spread, and enhanced penetration in deeper tumor tissue (Reprinted with permission from Ref. [137]. 2021, Jianqin Yan).

Additionally, Yang et al. [25] reported the obtaining of a redox-responsive drug delivery system based on a hyaluronic acid (HA)-grafted polymer loaded with DOX. The HA is conjugated with folic acid (FA) through a reduction-sensitive disulfide linkage in order to design an amphiphilic polymer (HA-ss-FA). Further, cystamine (CYS) was used as a cross-linking agent to link HA and FA. The DOX release was studied in a phosphate buffer solution (PBS), at physiological pH and temperature, in the presence of GSH, in order to simulate the environment of the tumor cells and it was observed that the presence of GSH influenced a faster release of DOX because of the disruption of the disulfide bond in the HA-ss-FA molecules in the reductive environment. Pandey et al. [133] presented a dual-stimuli-responsive drug-delivery system based on micelles for cancer therapy using Doxorubicin. The micelles are represented by an amphiphilic biocompatible miktoarm star copolymer comprising two hydrophobic poly(ε-caprolactone) (PCL) blocks, a short poly(propargyl glycine) middle block, and a hydrophilic glycopolypeptide (GP) block containing galactose units for targeting liver cancer cells and were tested for responsiveness via two stimuli, such as redox and enzymes. It was observed that with a higher concentration of GSH, the release of DOX was increased, and in the case of the absence or lower concentration of GSH in the media, the release was radically decreased probably due to the presence of a few lightly cross-linked micelles. Additionally, the TEM images showed that after 24 h, the obtained micelles showed aggregation, and after 48 h the micelles disappeared in the presence of GSH treatment showing that the micellar assembly is only rattled by GSH-mediated cleavage of disulfide bonds. A multifunctional dual-responsive drug delivery system for targeting tumor therapy based on hollow mesoporous nanosilica loaded with Doxorubicin was proposed by Huang et al. [73]. Cytochrome C (CytC) was used as a sealing agent for mesoporous nanosilica and also as a mediator of apoptosis by recruiting and activating caspase once it is released from the cell mitochondria to the cytoplasm via conjugation with Apoptotic protease activating factor-1 (Apaf-1) in the presence of Deoxyadenosine triphosphate (dATP) which is a nucleotide used for DNA synthesis as a substrate in DNA polymerase [134,135]. In addition, the usage of lactobionic acid (LA) was utilized as a targeting agent, especially for HepG2 cells due to the particular ligand binding to the asialoglycoprotein receptor (ASGP-R) of HepG2 cells. The purpose of this dual-responsive drug delivery was to create a special delivery of DOX in the presence of glutathione (GSH) and acidic pH in the tumor microenvironment. The drug-release results showed that an increased release was observed in the case of an acidic environment and in the presence of a higher concentration of GSH via the simultaneous breakage of disulfide bonds and disassociation of boronated ester bonds of the system, which can lead to cell apoptosis and tumor growth inhibition [73]. Saedi et al. [136] described a dual-sensitive drug delivery (redox and pH-sensitive) based on a folate-modified star-like amphiphilic copolymer based on castor oil for DOX. The drug release was studied in PBS (pH = 7.4, 2 μM GSH) and ABS (pH = 5.5), with or without 10 mM GSH, and the results showed that at pH 7.4 the release of DOX was slow and inefficient, but at pH 7.4 and 2 μM GSH, the release was approximately 20% and by increasing the concentration of GSH (10 mM GSH), the release was increased up to 39%. The acidic condition also favors the release, such as when at pH 5.5 and 10 mM GSH, the biggest release of DOX was observed. Yan et al. [137] described polyethyleneimine (disulfide cross-linked PEI, PSP)/tetrahedral DNA (TDNs)/Doxorubicin (DOX) nanocomplexes (NCs)-based redox-responsive drug delivery system. Figure 9 details the gradually disassembled drug delivery system through the breakage of the disulfide when it interacts with the intracellular high concentration of GSH at the tumor site. Further, the disassembled DDS penetrated the tumoral tissue, improving the therapeutic efficacy. An increase in the release was observed in the presence of an acidic environment and GSH (50%) due to cleavage of the disulfide linkages in the high concentration of GSH.

### 2.4. Enzyme-Responsive Drug Delivery System

In recent years, many intelligent systems have been studied for the release of Doxorubicin based on enzyme responsiveness [138,139,140,141]. Enzymatic-responsive drug delivery systems represent a very promising category, due to the fact that changes in the expression can be found in tumor cells of specific enzymes, such as proteases, phosphatases, and glycosidases, which can be very easily targeted by enzyme-mediated drugs’ release [130]. The enzyme-responsive drug delivery systems have ester bonds in their composition or the peptide structure that can be degraded by various enzymes specific to inflammation of the tumor location [142,143]. The main properties of enzyme-responsive drug delivery systems are biorecognition, selectivity, and catalytic efficacy [142]. Generally, enzymatic-responsive drug delivery systems are obtained from peptide hydrogels, polymers and polymer conjugates, and polymeric nanoparticles, but also from mesoporous silica nanoparticles, metal nanoparticles, and semiconducting nanoparticles [144,145,146]. Enzyme-responsive polymers are used to incorporate the therapeutic agent, and in the presence of the enzyme found in the body, to release the therapeutic agent in a targeted way [147]. The most widely used enzymes for drug delivery systems are the hydrolases, including proteases, lipases, and glycosidases, due to the simple design requiring the attachment of bioactive moieties to the carrier through enzyme cleavable unit [146]. Proteases are enzymes that break down the peptides at the level of amino acids, being involved in many physiological processes such as tissue remodeling, wound healing, and tumor invasion [148]. An overexpression of proteases has been associated with cancer, so proteases can be used to allow for the selective activation of smart drug delivery platforms [149]. Yildiz et al. [74] reported core–shell nanoparticles based on amphiphilic copolymers poly(lactic-co-glycolic acid)-b-poly-l-lysine and poly(lactic acid)-b-poly(ethylene glycol) for Doxorubicin-loaded protease-activated drug delivery systems. The cytocompatibility was evaluated on MDA-MB-231 breast cancer cells and a significantly reduced cell viability was observed at drug concentrations of 0.10 µM.

Lipase, such as phospholipase, are enzymes that hydrolyze fats. However, in this case, it has also been observed that it can play the role of a pathological indicator for various conditions, such as many kinds of cancers and other conditions such as thrombosis, congestive heart failure, inflammation, neurodegeneration, and infectious pathogens [148].

Another enzyme intensively studied in the enzyme-responsive drug delivery field is azoreductase, which is an enzyme produced by micro-organism species generally present in the colon [148].

Another studied enzyme is azoreductase, which is a reductase enzyme extensively studied in the case of liver cancer, so that Medina et al. [75] reported the development of enzyme-activated nanoconjugates for the treatment of liver cancer through the release of DOX by using L1-L4 azo-linkers to conjugate a generation of 5 of poly(amidoamine) dendrimer and designed to be able to bind to hepatic azoreductase enzymes. The obtained enzymatic-responsive system was tested on Hep G2 and Hep 3B cells. The results showed a non-toxicity for cardiomyocytes, comparing with the silenced toxicity after the classical administration of DOX at the same concentration and are readily cleavable by intracellular azoreductase enzymes proven to be effective in killing liver cancer cells, having IC_50_ value similar to free DOX. Sun et al. [150] reported an NTR-responsive 4-nitrobenzyl group, hydrophobic AIE, tetraphenyl ethylene (TPE), and polyethylene glycol hydrophilic moieties for DOX drug delivery trough nitro reductase (NTR)–catalyzed. It was reported that 4 HeLa and HEK 293T cells were used in order to evaluate the cytotoxicity. The TNP-based drug delivery system showed that the DOX release was possible in the presence of NADH due to high sensitivity and selectivity to NTR, leading to the breakdown of the micelles and DOX release.

**Figure 10:**
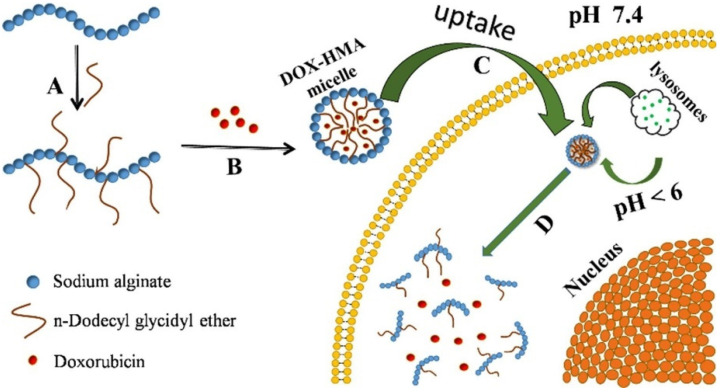
Schematic of synthesis and stimuli-responsive release of DOX-HMA micelles: (**A**) dodecyl glycidyl ether-modified alginate; (**B**) DOX encapsulated into HMA micelles; (**C**) DOX-loaded HMA micelles accumulated into tumor cells; (**D**) intracellular stimuli-responsive degradation and drug release. (Reprinted with permission from Ref. [152]. 2020, Xue Gao).

The main disadvantage of enzyme-responsive drug delivery systems is the release of the therapeutic agent before reaching the target area due to possible exposure to an enzyme trigger, or a closely related enzyme could release the load prematurely. This limitation can be overcome by obtaining a release system sensitive to dual stimuli, with a component sensitive to the pH variation, which favors a much more targeted release [151]. Gao et al. [152] reported a dual-responsive drug delivery based on hydrophobic-modified sodium alginate. In vitro cellular uptake and cytotoxicity were tested on HepG2 or Hela cells, and the results showed had good growth inhibition effects on HepG2 cells or Hela cells and also a slow release effect was shown. In Figure 10, the schematic synthesis and stimuli-responsive release is shown. This dual-stimulus drug delivery system is capable of releasing DOX in a much more targeted manner, due to lysosomal enzyme which is present in all mammalian cells.

## 3. External Stimuli-Responsive Drug Delivery Systems

### 3.1. Magnetic-Responsive Drug Delivery Systems

The extensively studied external stimuli-responsive drug delivery systems for cancer are the magnetic-responsive drug delivery systems. These kinds of smart drug-delivery systems can be achieved only if the nanocarrier possesses a strong magnetic property and can be employed by an applied magnetic field [153]. The first time was reported in 1980 by Kenneth et al. where the authors proposed the in vitro release of active Adriamycin via the utilization of magnetic-responsive albumin microspheres [154]. The magnetic-responsive DDS is able to be a selective target of antitumoral agents without disturbing the reticuloendothelial system (RES) in which an external magnetic field is applied to increase the drug concentration at the tumor site after administration of magnetic particles [155]. The mechanism of the magnetic drug-delivery system is that the therapeutic agent is linked to/or encapsulates magnetic nano/microparticles. Once attached, the magnetic-responsive DDS is injected into the bloodstream as a biocompatible ferrofluid and applied to a magnetic field in order to direct the DDS to the targeted tissue [156]. The magnetic susceptibility allows nuclear magnetic resonance (NMR) observation, tracking, and quantification of the antitumoral agent to the targeted tumoral tissue [157]. The most utilized magnetic-responsive nanocarriers are based on iron, cobalt, nickel, metallic oxides, mesoporous silica, calcium silicates, liposomes, and polymers [24]. Magnetic-responsive drug delivery systems consist of magnetic core-shell and polymer coatings [158]. In addition to the properties of the targeted release of the active substance, magnetic-responsive DDS have properties in diagnosis, being considered as theragnostic delivery systems combining imaging agents and effective therapeutic drugs [159].

**Figure 11:**
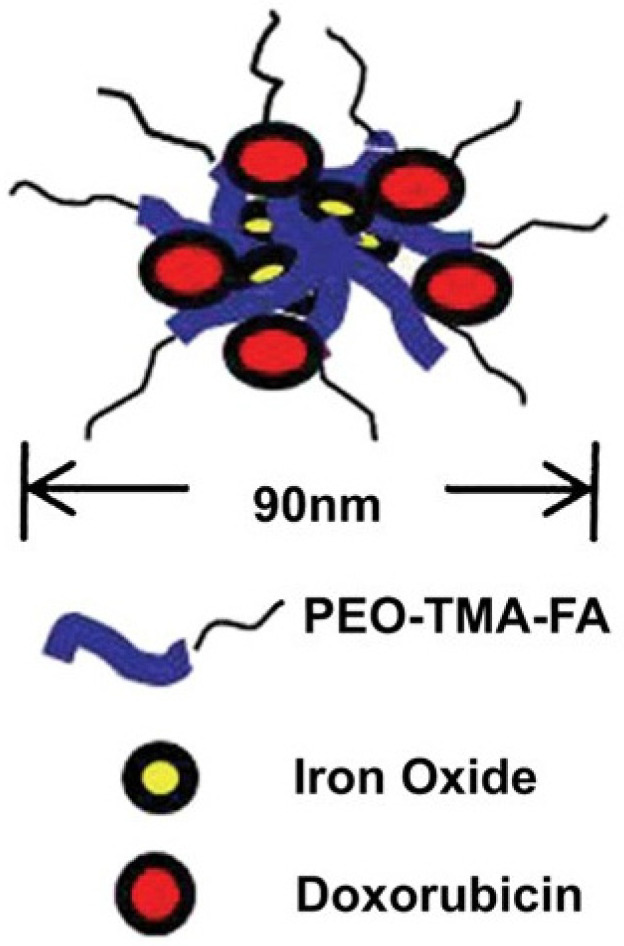
Structure of DDS based on PEO-TMA-FA, Iron Oxide and DOX (Reprinted with permission from Ref. [76]. 2010, Jin Hee).

In the last few years, many research papers based on developing a magnetic-responsive carrier have been published [160,161,162,163]. Jeon et al. reported the obtaining of smart porous nanoclusters used as potential drug delivery for DOX in liver cancer therapy via transcatheter intra-arterial infusion, which is a widely used medical technique based on image-guidance using X-ray angiography performed for inoperable liver tumors [164]. Superparamagnetic iron oxide nanoparticles (SPIONs) are frequently used in the production of magnetic-responsive drug delivery systems, that in comparison with ferromagnetic-based materials which are permanently magnetized, these types of materials are magnetized only in an applied field [161]. SPIONs are constituted through iron oxide cores (magnetite (Fe_3_O_4_) and/or maghemite (gFe_2_O_3_) nanocrystals) which could be targeted via application of an external magnetic field [165,166]. Li et al. presented the development of core-shell drug delivery based on SPIONs and DOX for dual purposes (tumor targeting and MRI diagnosis) [69]. In addition, superparamagnetic iron oxide (SPIO) is commonly used as an MRI contrast agent in order to evaluate or diagnose liver cancer [76,166]. Maeng et al. [76] proposed a magnetic-responsive drug delivery system based on poly(ethylene oxide)-trimellitic anhydride chloride-folate (PEO-TMA-FA), Doxorubicin (DOX), superparamagnetic iron oxide (Fe_3_O_4_) and folate, for liver cancer. The efficacy of the delivery system was demonstrated by in vivo tests on rats and rabbits with liver cancer, compared with the administration of DOX and a commercial liposome-based drug, DOXIL. In Figure 11, the authors graphically represent the model of the release system obtained for a better understanding of its structure. It can be seen that the core consists of iron oxides, and the core is coated in PEO-TMA-FA, and DOX is attached to the polymer surface. Folic acid (FA) was used to enter the receptor-expressing cancer cells through folate receptor-mediated endocytosis [167,168] and a special delivery of the targeting agent due to increased concentration of folate receptor (FR) in tumoral tissues. First, it was shown that there is a high level of FR in the case of liver cancer, compared to healthy tissue, and later, the effectiveness of DOX release was demonstrated using the DDS obtained. The in vitro test that investigates the anticancer effect shows that the obtained DDS was able to inhibit the proliferation of liver cancer cells more than DOXIL. Additionally, the in vivo test shows a significantly decreased tumor volume and did not produce visible side-effects [76].

**Figure 12:**
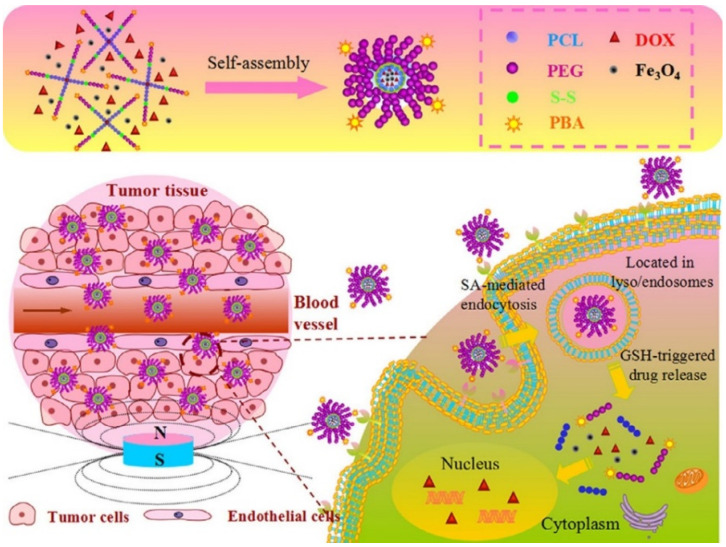
Schematic illustration of the fabrication of DOX-loaded magnetic star-shaped polymer micelles and the procedure to selectively target the SA-positive HepG2 cells by the dual-targeting, and the fast response by GSH on drug release in the cytoplasm (Reprinted with permission from Ref. [77]. 2016, Zhaomin Tang).

Further, for an improved release, an attempt is made to combine two or more multiple stimuli, as in the case of Tang et al. [77], who developed a release system so sensitive to the magnetic stimulus as to direct the path of the therapeutic substance to the target tissue, as well as being sensitive to the reducing environment, so that at the moment of interaction with the tumor tissue the release is performed through the obtaining of star-shaped magnetic micelles based on self-assembly poly(ethylene glycol) (PEG)-poly(e-caprolactone) (PCL) copolymers with magnetic iron oxide nanoparticles (Fe_3_O_4_) and DOX encapsulated into the hydrophobic core of the micelles (Figure 12) [77].

**Figure 13:**
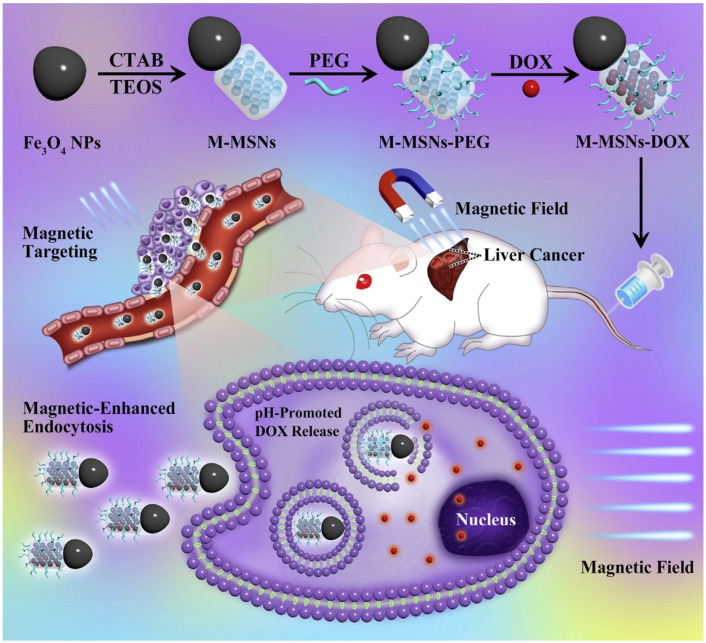
Schematic illustration of the synthetic procedure for the Janus M-MSNs-DOX nano-bullet and application for pH-promoted drug release after magnetic-enhanced endocytosis in cancer cells, as well as magnet targeting liver cancer chemotherapy in vivo (Reprinted with permission from Ref. [169]. 2016, Dan Shao).

Shao et al. [169] developed magnetic nanoparticles as delivery carriers based on magnetic Fe_3_O_4_ and a body of mesoporous SiO_2_ containing Doxorubicin (DOX) as “nano-bullets” (Figure 13). The loading efficiency was reported as being 60% and the drug-loading content was 20%. It was observed that the drug retention is increased by the magnetic field, and the acidic pH increased the release rate of DOX (50% of DOX is released at pH 5.5 in 24 h in comparison with less than 5% at pH 7.4 in 24 h). Cheraghi et al. [170] proposed cobalt ferrite magnetic nanoparticles for antitumoral drug release. The treatment with lemon juice with different volumes was used as a chelating agent. It was observed that an increase in the lemon juice contents can partially replace the Fe^3+^ ions with Fe^2+^ ions, reducing the super-exchange interaction between the magnetic atoms at octahedral and tetrahedral sites (Fe^3+^-O-Fe^3+^), leading to a drop at the net magnetic moment. Additionally, the obtained drug delivery was dual-responsive, such that the pH values influenced the drug release along with the magnetic response, leading to the best release of DOX by more than 42% at pH 5.4 in 75 h. Li et al. [171] employed polyethylene glycol (PEG)-functionalized γ-Fe_2_O_3_ particles (γ-Fe_2_O_3_/PEG) as a dual drug delivery of DOX. After applying an AMF (45 kA/m and 186 kHz), the cumulative releases were increased to 32, 45, 55, and 63% under the pH values of 7.2, 6.5, 6.0, and 5.5. Further, a higher release of DOX was observed in acidic pH due to higher DOX solubility in the acid environment. Ren et al. [154] presented magnetite nanoparticles and graphene oxide (GO/Fe_3_O_4_) drug delivery loaded with Doxorubicin hydrochloride via a chemical precipitation method. The release was increased by the addition of a magnetic field by inducing a temperature increase through magnetic hyperthermia. The challenges for using magnetic-responsive drug delivery systems are: the potential toxicity of metallic nanoparticles, so that in many studies the accumulation of various magnetic nanoparticles in various vital organs has been reported [172,173]; a fairly large magnetic field is needed; and it is very difficult to focus the alternating magnetic field [39,149,161].

### 3.2. Photo-Responsive and Photothermal-Responsive Drug Delivery Systems

Photo-responsive drug delivery systems have been an attractive option for the controlled release of drugs using light sources, such as ultraviolet (UV), visible, and near-infrared (NIR) light [174]. The photo-responsive drug delivery systems have numerous advantages, such as being safe, minimally invasive, and a tissue-selective treatment for cancer therapy [175,176,177,178]. Most commonly used light-responsive agents are reversible such as azobenzene, spiropyran, dithienylethene, and diazonaphthoquinone, and irreversible, such as o-nitrobenzy, pyrenylmethyl, and coumarin [179]. These light-responsive agents are capable, when irradiated with light radiation, to disturb the bilayer membrane of the corresponding polymersomes (artificial vesicles enclosing an aqueous cavity) resulting in the self-assembly of the amphiphilic copolymer, which reorganizes into smaller polymers leading to the drug release [179,180]. Boruah et al. [181] reported photo-responsive drug delivery systems based on liposome-azobenzene nanocomposite for DOX. The results showed a drug release of 77.33% in comparison to pure vesicles’ release of ~3.3%. Zhou et al. [182] developed azo-bearing diblock copolymers as a photo-responsive and pH-responsive drug delivery system for DOX. The results showed excellent cytocompatibility on human breast-cancer cell (MCF-7) and a burst release was observed in the first 2 h and after 10 h 90% was released. Additionally, when spiropyran (SP) is under the action of irradiation with a specific wavelength of light (200–400 nm), a ring-opening occurs through a cis-trans isomerization, to zwitterionic merocyanine (MC) [39,183]. Chen et al. [180] reported a multi-sensitive drug delivery system, including photo-responsive stimuli based on poly(acrylic acid-co-spiropyran methacrylate) crosslinked by disulfide-containing N,N-bis(acryloyl)cystamine for DOX release. After irradiation, the addition of spiropyran lead to the hydrophobic SP in nanogels isomerized to the hydrophilic MC and then the nanogel swelled, resulting in a disruption of nanogel due to the oxidative scission of the disulphide crosslinkers, facilitating the drug release. The limitations of UV-responsive drug delivery systems are poor tissue penetration and the damaging effects on healthy tissues [184]. As a solution for these limitations, different near-infrared (NIR) or visible light-responsive materials have been developed [6,185,186].

**Figure 14:**
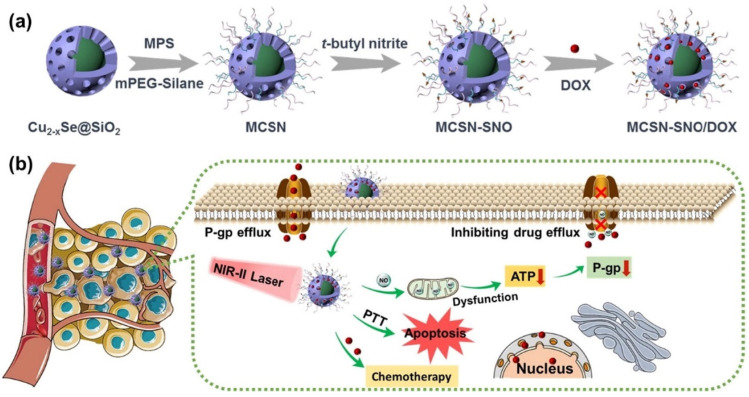
Schematic illustration of (**a**) the synthetic procedure of MCSN-SNO/DOX and (**b**) the process of photothermal-responsive NO gas release for overcoming multidrug resistant tumor by combination therapy (Reprinted with permission from Ref. [198]. 2021, Youyou Huang).

Photothermal-responsive drug delivery systems are based on external light, having many advantages, such as a noninvasive nature, simplicity of operation, good controllability over both wavelength and intensity, and high spatiotemporal resolution [6,187,188,189]. Photothermal therapy uses a photothermal agent that is stimulated by both specific band light and vibrational energy/heat release to selectively target tumoral tissue. Photo-responsive DDS are characterized by three main categories: (i) photothermal responsive DDS; (ii) photodynamic responsive DDS; and (iii) photoconversion-responsive DDS [6]. Drug delivery systems based on the photothermal response have a dual role, in cancer diagnosis and treatment [190]. The photothermal-responsive DDS has mandatory main components, such as chromophore which is able to absorb the applied energy and the corresponding wavelength and achieves an excited state, followed by the conversion of the absorbed light energy into thermal, and the second main component is the thermally responsive material that has an increased sensibility at temperature variation [191,192]. The most used materials for this kind of DDS are gold nanoparticles [193,194,195] and NIPAAm [196]. Ji et al. [197] reported a new drug-release system based on gold nanocages (AuNCs) as the photothermal core, Hyaluronan (HA) as the targeting ligand, P(NIPAM-co-Am) (PM) as the thermally responsive copolymers, and Doxorubicin as the therapeutic agent for liver cancer therapy. It was shown that in vitro results demonstrate that the obtained DOX/AuNCs-PM-HA has increased anticancer activity and in vivo photoacoustic tomography imaging shows effective tumor targeting. Modification by conjugation of HA to the outer surface of the delivery vector improved this delivery system targeting ability, leading to a higher drug concentration at the tumor site, reduced toxicity and side effects of DOX, and an improved curative effect. Huang et al. [198] proposed mesoporous core–shell structured nanocomposites (MCSN) of Cu2-xSe@SiO (Figure 14). It was observed that the composites displayed a concentration-dependent temperature change under laser irradiation. The release rate was reported as 42% at pH = 4.6 in 6 h, without irradiation and 55.4% at pH = 4.6 after adding NIR laser irradiation, and became slow without laser irradiation. Chen et al. [199] proposed a photothermal drug delivery based on gelatin. After irradiation, an increase was reported in local temperature, up to 55.9 °C. Li et al. [200] described an iron-oxide-based transdermal drug delivery with high photothermal conversion efficiency and stability, efficient cellular uptake, synergistic cancer cell-killing effect, and enhanced percutaneous permeability in vitro.

### 3.3. Electric-Responsive Drug Delivery Systems

Another studied type of external stimuli drug-delivery system is based on electrical stimulation. Electrical stimuli were used in order to generate the release of the therapeutic agent by conductive polymeric bulk materials and implantable electronic delivery devices [201]. Conductive polymers are used in many biomedical applications, such as biosensors, in nerve tissue regeneration, and drug delivery systems [202,203,204,205], due to their unique electrical and optical properties, very similar to metals and inorganic semiconductors [206]. The main advantages of conductive polymers are that their chemical, electrical and physical properties can be tailored to the specific needs, and that they have metal conductivity properties, but also have the flexibility of polymers [202,207,208]. The most-used conductive polymers to obtain electric-responsive drug delivery systems are Polyaniline (PANI) [209], polypyrrole (PPy) [210], and poly(3,4-ethylenedioxythiophene) (PEDOT) [211]. The conductive polymers are used to customize the drug delivery systems, so that when an electric field is applied, it can facilitate the release of the antitumor agent in the desired area. The accomplishment of this kind of conductive polymer can be influenced by the nature of the chosen dopant and the molecular weight of the antitumoral agent [212]. The main principle of the utilization of electrically responsive drug delivery systems is the application of a weak electric field over targeted tumoral tissue after the administration of electro-responsive drug carriers for controlled on-site drug release [213]. The major limitation of electric-responsive drug delivery systems is the fact that they are limited to topical or subdermal implants, due to the need to place electrodes in the polymer matrix [151].

### 3.4. Ultrasonic-Responsive Drug Delivery

**Figure 15:**
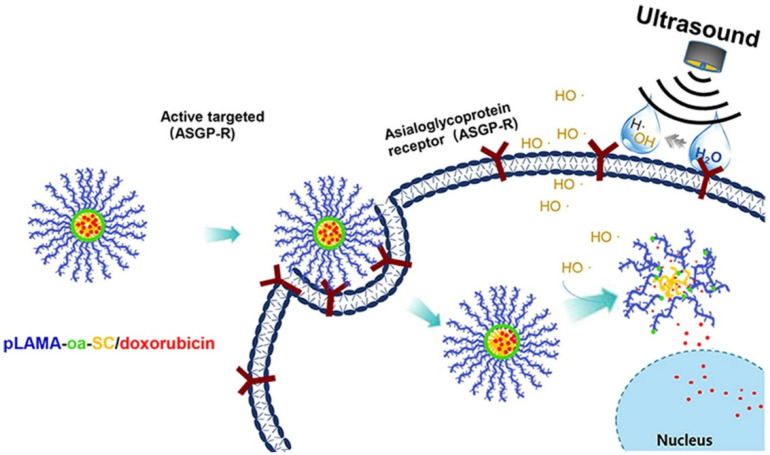
Schematic illustration of ultrasound-labile motif and hepatocellular carcinoma-targeted drug delivery system (pLAMA-oa-SC/Doxorubicin self-assembled nanostructures) for ultrasound-specified augmented cytotoxic potency to hepatocellular carcinoma (Reprinted with permission from Ref. [80]. 2019, Jingyun Wang).

Ultrasonic-responsive drug delivery is a less utilized system that is sensitive to external stimuli, but it can represent a promising trigger for stimuli-responsive drug delivery due to its amazing characteristics, such as the ability to non-invasively penetrate deeply into the tissue without damaging it [214,215]. The ultrasound waves are able to permeate the materials far deeper than light and has the advantage that there is no need for further functionalization of the biomaterials in order to introduce additional functional groups to increase the reactivity of the biomaterial, as the polymers heat up in seconds under exposure to ultrasound [163]. Ultrasounds are defined as mechanical waves with high frequencies (≥20 kHz) which are able to travel through a medium [214]. Generally, ultrasound waves have applications in different fields, such as in vivo imaging, physiotherapy, cosmetics and food industry [216]. In general, release systems based on ultrasound waves are obtained from polymers, in different forms, such as polymer-coated bubbles/emulsions, and polymer hydrogels [216,217,218,219,220]. It was reported that ultrasonic-responsive drug delivery systems are able to increase drug delivery, but also increase the therapeutic efficacy, and temporal release of drugs [221]. The use of ultrasound waves can induce thermal effects, mechanical effects, or radiation force, which can lead to the release of the active substance in a non-invasive, remote, and spatiotemporally controlled manner [222]. Kim et al. [79] developed Doxorubicin-loaded albumin nanoparticle-conjugated microbubble complex in an iodized oil emulsion as an ultrasonic-responsive drug delivery system for liver cancer. The delivery of DOX was possible due to the sonoporation effect induced by the cavitation of microbubbles. Wang et al. [80] reported poly(lactobionamidoethyl methacrylate)-based amphiphiles as ultrasonic-responsive drug delivery systems (Figure 15). The results showed that the accelerated release was attributed to the ultrasound-induced cleavage of -oa- linkage, leading to intracellular drug release from delivery system increasing the cytotoxic potency at the targeted cells. Zhou et al. [223] reported chitosan nanobubbles for ultrasound-mediated targeted delivery of Doxorubicin. The results showed an near-instantaneous cellular entry of DOX due to sonoporation. In addition, Wu et al. [224] reported that sonoporation inhibited tumor growth and the decrease in tumor weight was approximately 6.5-fold in comparison, without exposure to ultrasound irradiation. Gao et al. [81] developed ultrasound- and pH-responsive delivery of DOX based on alginate/chitosan stabilized perfluoro hexane nanodroplets. The in vitro and in vivo tests showed that DOX-loaded nanodroplets have a good accumulation and tumor-targeting in HepG2 tumors, resulting a growth inhibition of the tumoral tissue under the effect of ultrasound. A very important limitation of ultrasound-responsive drug delivery systems are represented by the possible tissue damage caused by heating and by irreversible pore formation in cell membranes [39,225,226].

## 4. Conclusions and Future Perspectives

Chemotherapy is the most widely used technique for treating liver cancer. Disadvantages of this method of treatment are numerous side effects, lack of therapeutic agent targeting, and the solubility and stability of anticancer drugs. Furthermore, the most commonly used antitumor agent in the treatment of liver cancer is Doxorubicin. A possible solution for the limitation of the conventional chemotherapy is by using intelligent controlled-release systems, capable of releasing the antitumor agent in a controlled manner in the presence of stimuli. In this review, we present and summarize both internal stimuli-responsive drug delivery systems, such as redox, pH and temperature variation, and external stimuli-responsive drug delivery systems, such as the application of magnetic, photothermal, and electrical stimuli, for the controlled release of Doxorubicin in liver cancer therapy. Furthermore, drug delivery systems sensitive to multiple stimuli are an attractive subject to researchers due to their increased targeted efficiency. The studies of stimuli-responsive drug delivery systems of Doxorubicin are still at an incipient stage as they require multiple preclinical evaluations of their biocompatibility and toxicity. Future trends can be divided into two main directions. The first one is related to ever increasingly precise materials and technologies that will allow precise amounts of DOX to be discarded at the tumor site with minimum impact for the rest of the organism. This would be possible due to higher accessibility at an industrial scale to targeted drug-delivery systems based using as target molecule the concentrations of tumor marker in the blood (especially alpha-fetoprotein) and also by the possibility to concentrate the drug delivery system at the tumor site. Supramolecular architectures that incorporate monoclonal antibodies for tumor markers are an elegant solution to this problem, but their cost remains prohibitive at the moment and the actual synthesis routes make this plan utopian. Another future direction would be represented by coupling the DOX-targeted drug delivery with another medical procedure—hemodialysis. A large number of patients with chronic kidney disease will develop a form of liver cancer due to the accumulation of creatinine, urea, and uric acid. Hemodialysis membranes that incorporate targeted drug delivery systems for liver cancer would increase the life quality of patients that suffer from both medical conditions.

## Figures and Tables

**Table 1:** Summary of featured stimuli-responsive polymers.

Stimuli	Smart-Drug Delivery System	Results	Ref.
pH	POEAd-g-LA-DOX micelles	pH changes promote chemical and physical modifications resulting drug release	[61]
pH	HA-hyd-DOX	pH changes promote hydrazone bonds disintegrated resulting in drug release	[62]
pH	S(HA–GA/HA–His)	pH changes promote the swelling of the core, followed by the DOX release.	[63]
pH	CS-NSA/A-HA/DOX	Drug release at pH5.5	[64]
pH	CEC-PEGDA	pH changes promote chemical and physical modifications resulting in drug release	[65]
pH	folic acid-modified and zeolitic imidazolate framework (ZIF)	pH changes promote chemical and physical modifications resulting in drug release	[66]
Temperature	β-CD-g-(PNIPAAm-b-POEGA)x/DOX	Temperature greatly influences the release of DOX	[67]
Temperature pH	Graphene nanosheets-poly(N-isopropylacrylamide)-polyethylenemine	Drug release at pH 5.5 and above LCST	[68]
Temperature pH Magnetic	magnetic iron oxide (MIO) nanoparticles functionalized with Pluronic F127 (PF127) and branched polyethylenimine (bPEI)	pH and temperature greatly influence the release of DOX	[69]
Temperature Magnetic	phosphatidylglycerol (DPPG2) -d (MR-HIFU)	Release at 42 °C due to the melting temperature	[70]
Redox	anti-carbonic anhydrase IX antibody (A-CAIX Ab)	Release due to disulfide linkages	[71]
Redox	(HA-Cyst-GA)	Release due to the presence of reductive stimulus	[72]
Redox	(HA-ss-FA)	Release due to the presence of GSH	[25]
Redox pH	Phenylboronic acid-modified hollow silica nanoparticles	Release due to the presence of GSH and pH variation	[73]
Enzyme	poly(lactic-co-glycolic acid)-b-poly-l-lysine and poly(lactic acid)-b-poly(ethylene glycol)	Release due to protease-mediated cleavage	[74]
Enzyme	poly(amidoamine) dendrimer	Bind to hepatic azoreductase enzymes via a NADPH-dependent mechanism	[75]
Magnetic	poly (ethylene oxide)-trimellitic anhydride chloride-folate (PEO-TMA-FA),	Significantly decreased tumor volume without visible side effects	[76]
Magnetic Redox	poly(ethylene glycol) (PEG)-poly(e-caprolactone) (PCL) copolymers with magnetic iron oxide nanoparticles (Fe 3 O 4)	Selectively target the SA-positive HepG2 cells by magnetic and enzymatic stimuli	[77]
Photo-responsive-UV Redox pH	poly(acrylic acid-co-spiropyran methacrylate) crosslinked by disulfide-containing N,N-bis(acryloyl)cystamine	Released upon the stimulation of light, pH, and DTT.	[78]
Ultrasonic	albumin nanoparticle-conjugated microbubble complex	Drug release via sonoporation	[79]
Ultrasonic	poly(lactobionamidoethyl methacrylate)-based amphiphiles	Accelerated release was attributed to the ultrasound-induced cleavage of -oa- linkage	[80]
Ultrasonic	alginate/chitosan stabilized perfluoro hexane nanodroplets	Good accumulation and tumor-targeting in HepG2 tumors	[81]

## Data Availability

Not applicable.
